# Demographics and Clinical Presentations of Patients Visiting the Emergency Department During the Holy Month of Ramadan: A Three-Year Retrospective Study in a Muslim-Majority Country

**DOI:** 10.7759/cureus.40373

**Published:** 2023-06-13

**Authors:** Imad M Khojah, Mohammed A Alsubaie, Saeed A Alhudaifi, Anas S Alyazidi, Maha K Alghamdi, Abdullah A Bakhsh, Waddaa R Reda

**Affiliations:** 1 Department of Emergency Medicine, King Abdulaziz University Hospital, Jeddah, SAU; 2 Department of Emergency Medicine, Faculty of Medicine, King Abdulaziz University, Jeddah, SAU; 3 Medicine and Surgery, King Abdulaziz University, Jeddah, SAU; 4 Department of Internal Medicine, King Faisal Specialist Hospital and Research Centre, Jeddah, SAU

**Keywords:** triage protocols, saudi arabia, holey ramadan, religious fasting, emergency medical service

## Abstract

Background and objective

Ramadan is the Muslim's holiest month; it is a time when believers engage in special practices that include fasting from dawn till dusk and making cultural and dietary modifications in their everyday lives. The impact of Ramadan on human activity, sleeping patterns, and circadian rhythms of hormones have been addressed in the literature. Fasting, which constitutes the main pillar of practices during Ramadan and lasts from sunrise to sunset, can significantly affect common health conditions, leading many to seek medical care in the Emergency Department (ED). Hence, it is important to understand the pattern of ED visits and understand the impact caused by fasting during this holy month in a Muslim-majority country. In light of this, this study aimed to gather new insights into the pattern of ED visits during Ramadan at a busy tertiary care center in the period from 2019 to 2021.

Methods

This study was conducted by reviewing the hospital health information system to gather relevant information in May 2022. Data of patients who visited the ED during Ramadan were collected, as well as during a month prior to and after Ramadan for the purpose of comparison. Sociodemographic characteristics and clinical profiles were collected for analysis.

Results

The total number of ED visits in the three months of Ramadan during the study period (three years) was 33,142, all of which were included in our analysis. Sociodemographic data were analyzed for patients who visited the ED during the month of Ramadan and the two lunar months that precede and succeed Ramadan (Shaban and Shawal). Fever was the most common complaint (16.5%), followed by abdominal pain (14%). When analyzing the findings based on patient age groups, fever was found to be the most prevalent complaint in both adults (15.6%) and pediatric patients (34.4%). Of the total ED patient visits, 7,527 patients were admitted for further care, and 197 patients deceased.

Conclusion

Our study findings illustrate the change in ED visit patterns during the month of Ramadan in a Muslim-majority country. Also, the type of complaints was affected significantly due to the ongoing coronavirus disease 2019 (COVID-19) pandemic during the study period. The outcomes in patients reflected substantial progress and outcomes in the ED. These findings highlight that analyzing ED data can help provide accurate information that can be used to help modify/adjust the quality of services provided in the ED. However, these modifications may affect all hospital facilities, not just the ED.

## Introduction

The Islamic Hijri calendar consists of 12 lunar months, and each of them lasts for 29-30 days. Ramadan is the ninth month in the Hijri calendar and is considered the holiest month by Muslims. According to the tenets of Islam, all healthy Muslims who have reached the age of puberty are required to fast from sunrise to sunset during Ramadan, by eschewing even drinking water [1]. Fasting during Ramadan could lead to variations in routine activities of daily life, sleeping patterns, and circadian rhythms of hormones [2]. Hence, immense changes in daily physical habits and activities have been observed among Muslims during Ramadan, which could lead to changes in cognitive function [3], mood swings, and weight gain [4,5].

The practice of fasting significantly affecting common health conditions has been reported in the Saudi population, including metabolic disorders, leading to an increase in the frequency of hospital visits due to various medical complications [6]. Furthermore, aggressive behavior that some people are prone to during Ramadan can result in major traumatic incidents, most notably road traffic accidents. Road traffic accidents are a major burden on the healthcare system in Saudi Arabia, particularly during Ramadan [7]. This has been reported in other Muslim-majority countries as well, including Pakistan, where 96.47% of the population is Muslim according to their 2017 national census [8]. Moreover, another study has illustrated the changes in health patterns that fasting is associated with respect to specific medical conditions, mainly in diabetic and hypertensive patients [9]. Nonetheless, there is scarce literature from the Middle East, especially Saudi Arabia, on the pattern of Emergency Department (ED) visits analyzing the patients' demographic characteristics and other variables during Ramadan. Hence, in this study, we aimed to gain and present a novel understanding of the pattern of ED visits at a busy tertiary care center during the months of Ramadan in the period from 2019 to 2021.

## Materials and methods

Ethical consideration

This study followed the ethical guidelines for retrospective studies, and it was reviewed and approved by the Unit of Biomedical Ethics Research Committee at King Abdulaziz University with reference number 209-22. The study was conducted according to the World Medical Association Declaration of Helsinki, and informed consent was waived due to the nature of the study design.

Study design and setting

This retrospective study was conducted by reviewing the Phoenix electronic medical records and the health information system in May 2022. The targeted population consisted of patients of all ages who visited the ED at King Abdulaziz University Hospital (KAUH), Jeddah, Saudi Arabia. KAUH is a tertiary hospital and is part of a government-funded multispecialty healthcare system in Saudi Arabia. The study analyzed ED visits of patients during the period from 2019 to 2021. Data of patients who visited the ED during the month of Ramadan and the two lunar months that precede and succeed Ramadan (Shaban and Shawal) were analyzed. Ramadan is the ninth month in the Hijri calendar, which is based on lunar observation; thus, the month of Ramadan during the study period corresponded to the following dates in the Gregorian calendar: 6 May 2019-3 June 2019, 2 April 2020-1 May 2020, and 13 April 2021-12 May 2021. No restrictions were applied in terms of the inclusion criteria, and all patients who visited the ED during the study period were included. 

The Australasian Triage Scale (ATS) was used to determine the triage level categorization in our study, which was carried out in accordance with local standardized triage protocols and guidelines set by our hospital. These protocols classify patients based on how urgently they need medical care in order to ensure patient safety and effective resource allocation.

Statistical analysis

The following data items were retrieved for statistical analysis: sociodemographic characteristics, such as gender, age, and nationality; and clinical features, including patients' triage priority, ED section, and medical complaints. Categorical variables were presented as frequencies and percentages. Data management and analysis were performed using IBM SPSS Statistics version 25 (IBM Corp., Armonk, NY).

## Results

Demographics and clinical characteristics

A total of 33,142 patients visited the ED in the selected three months during the study period (three years), all of whom were included in our analysis. Sociodemographic data were analyzed for patients who visited the ED during the month of Ramadan and the two lunar months that precede and succeed Ramadan (Shaban and Shawal) (Table 1).

**Table 1:** Demographic and clinical profile of patients visiting ED in the three lunar months from 2019/1440H to 2021/1442H (n=33,142)

Variable	Shaban (n=10,809)	Ramadan (n=11,106)	Shawal (n=11,227)	Total
Gender, n (%)				
Male	4,860 (45)	5,068 (45.6)	4,932 (43.9)	14,860 (44.8)
Female	5,949 (55)	6,038 (54.4)	6,295 (56.1)	18,282 (55.2)
Age group, years, n (%)				
0–18	2,181 (20.2)	2,163 (19.5)	2,206 (19.6)	6,550 (19.8)
19–39	4,809 (44.5)	4,905 (44.2)	4,958 (44.2)	14,672 (44.3)
40–59	2,287 (21.2)	2,552 (23.0)	2,442 (21.8)	7,281 (22)
60+	1,532 (14.2)	1,486 (13.4)	1,621 (14.4)	4,639 (14)
Nationality, n (%)				
Saudi	7,659 (70.9)	7,290 (65.6)	7,878 (70.2)	22,827 (68.9)
Non-Saudi	3,150 (29.1)	3,816 (34.4)	3,349 (29.8)	10,315 (31.1)
Triage level, n (%)				
Priority 1 - resuscitation	164 (1.5)	169 (1.5)	171 (1.5)	504 (1.5)
Priority 2 - emergent	1,479 (13.7)	1,523 (13.7)	1,602 (14.3)	4,604 (13.9)
Priority 3 - urgent	5,180 (47.9)	4,803 (43.2)	5,017 (44.7)	15,000 (45.3)
Priority 4 - less urgent	3,729 (34.5)	4,365 (39.3)	4,286 (38.2)	12,380 (37.4)
Priority 5 - non-urgent	257 (2.4)	246 (2.2)	151 (1.3)	654 (2)
ED section, n (%)				
Adult	7,469 (70.0)	7,909 (71.3)	7,941 (70.7)	23,319 (70.412)
Pediatric	1,691 (15.6)	1,603 (14.4)	1,709 (15.2)	5,003 (15.1)
Ob/Gyn	1,649 (15.3)	1,594 (14.4)	1,577 (14)	4,820 (14.5)

Of the total sample, the majority (n=18,282, 55.2%) were female. Patients between the ages of 19 and 39 years were the predominant group with 14,672 (44.3%) visits, while patients who were 60 years and older were the least represented group with 4,639 (14%) visits. The majority of the patients were Saudi nationals (68.9%). Patients were categorized according to the severity of their condition/priority of care into five groups. Patients with Priority 3 (urgent) constituted the highest number of visits (n=15,000, 45.3%). Furthermore, patients were stratified into four categories according to the ED section they visited: the adult section had the highest number of visits (n=23,228, 70.1%), followed by the pediatric section with 5,003 (15.1%) visits.

Patient complaints 

**Figure 1:**
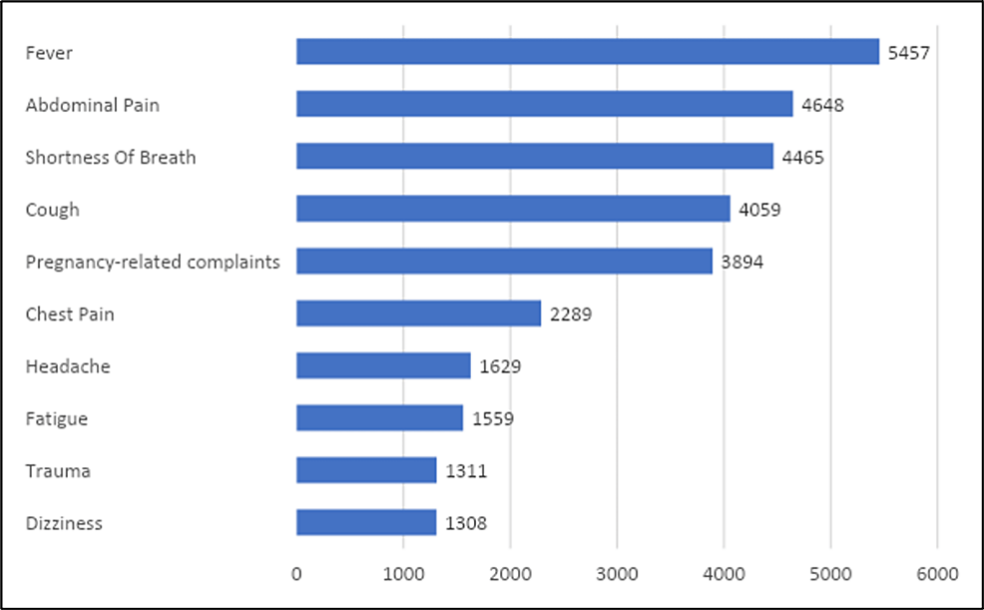
Top 10 most common complaints among patients who visited the ED in the three lunar months from 2019/1440H to 2021/1442H (n=33,142)

Figure 1 displays the top 10 most common complaints among patients visiting the ED. Fever was the most common complaint (16.5%), followed by abdominal pain (14%), shortness of breath (13.5%), cough (12.2%), pregnancy-related complaints (11.7%), chest pain (6.9%), headache (4.9%), fatigue (4.7%), trauma (4%), and dizziness (3.9%).

In Table 2, the most common complaints are presented in terms of their frequencies in each of the lunar months across the three-year study period. During the year 2019, the most frequent complaint during the month of Shaban was pregnancy-related (21.7%), followed by abdominal pain (19.6%), fever (15.3%), chest pain (9.9%), and shortness of breath (9.2%). During the month of Ramadan of the same year, the most frequent complaint was pregnancy-related (20.1%), followed by abdominal pain (19.7%), fever (13.5%), shortness of breath (10.2%), and headache (9.6%). As for the month of Shawal of the same year, abdominal pain was the most frequently reported complaint (18.8%), followed by pregnancy-related complaints (17.9%), fever (11.9%), vomiting (11.8%), and shortness of breath (10.4%).

**Table 2:** Most common complaints yearwise among patients who visited the ED in the three lunar months from 2019/1440H to 2021/1442H (n=33,142)

2019, n (%) (n=12,399)			2020, n (%) (n=10,015)			2021, n (%) (n=10,728)		
Shaban (n=4,479)	Ramadan (n=3,993)	Shawal (n=3,927)	Shaban (n=2,753)	Ramadan (n=3,614)	Shawal (n=3,648)	Shaban (n=3,577)	Ramadan (n=3,499)	Shawal (n=3,652)
Pregnancy-related complaints: 970 (21.7)	Pregnancy-related complaints: 803 (20.1)	Abdominal pain: 738 (18.8)	Pregnancy-related complaints: 887 (32.2)	Fever: 1,206 (33.4)	Fever: 1,275 (35.0)	Abdominal pain: 667 (18.6)	Fever: 636 (18.2)	Fever: 718 (19.7)
Abdominal pain: 880 (19.6)	Abdominal pain: 785 (19.7)	Pregnancy-related complaints: 701 (17.9)	Fever: 591 (21.5)	Cough: 1,145 (31.7)	Cough: 1,238 (33.9)	Fever: 555 (15.5)	Abdominal pain: 618 (17.7)	Abdominal pain: 678 (18.6)
Fever: 687 (15.3)	Fever: 540 (13.5)	Fever: 466 (11.9)	Cough: 522 (19.0)	Pregnancy-related complaints: 948 (26.2)	Pregnancy-related complaints: 947 (26.0)	Chest pain: 417 (11.7)	Shortness of breath: 458 (13.1)	Cough: 575 (15.7)
Chest pain: 445 (9.9)	Shortness of breath: 407 (10.2)	Vomiting: 465 (11.8)	Shortness of breath: 463 (16.8)	Shortness of breath: 621 (17.2)	Shortness of breath: 718 (19.7)	Pregnancy-related complaints: 417 (11.7)	Pregnancy-related complaints: 410 (11.7)	Shortness of breath: 543 (14.9)
Shortness of breath: 410 (9.2)	Headache: 383 (9.6)	Shortness of breath: 410 (10.4)	Abdominal pain: 412 (15.0)	Sore throat: 548 (15.2)	Abdominal pain: 504 (13.8)	Shortness of breath: 435 (12.2)	Chest pain: 398 (11.4)	Chest pain: 476 (13.0)

As for the year 2020, during the month of Shaban, pregnancy-related complaints and fever together constituted more than half of the reported complaints (32.2% and 21.5%, respectively). These complaints were followed by coughing (19.0%), shortness of breath (16.8%), and abdominal pain (15.0%). During Ramadan of the same year, fever was the most frequently reported complaint, accounting for 33.4% of the cases. This was followed by cough (31.7%), pregnancy-related complaints (26.2%), shortness of breath (17.2%), and sore throat (15.2%). During the month of Shawal of the same year, fever constituted 35.0% of the cases, followed by cough (33.9%), pregnancy-related complaints (26.0%), shortness of breath (19.7%), and abdominal pain (13.8%).

Finally, for the year 2021, during the month of Shaban, abdominal pain was the most frequently reported complaint (18.6%), followed by fever (15.5%), chest pain (11.7%), pregnancy-related complaints (11.7%), and shortness of breath (12.2%). As for the month of Ramadan, fever was the most frequently reported complaint (18.2%), followed by abdominal pain (17.7%), shortness of breath (13.1%), pregnancy-related complaints (11.7%), and chest pain (11.4%). Also, for the month of Shawal, fever was the most frequently reported complaint (19.7%), followed by abdominal pain (18.6%), cough (15.7%), shortness of breath (14.9%), and chest pain (13.0%).

Overall, the most frequent complaint was fever (17.4%), which was followed by abdominal pain (13.7%), shortness of breath (13.4%), cough (13.3%), pregnancy-related complaints (11.6%), vomiting (6.8%), sore throat (6.7%), chest pain (6.3%), headache (5.2%), and fatigue (4.6%), as shown in Table 3.

**Table 3:** Most common complaints overall among patients who visited the ED in the three lunar months from 2019/1440H to 2021/1442H (n=11,106)

Complaints	N (%)
Fever	1,931 (17.4)
Abdominal pain	1,526 (13.7)
Shortness of breath	1,486 (13.4)
Cough	1,473 (13.3)
Pregnancy-related complaints	1,286 (11.6)
Vomiting	756 (6.8)
Sore throat	748 (6.7)
Chest pain	699 (6.3)
Headache	578 (5.2)
Fatigue	506 (4.6)

Patient outcomes

The total number of ED patient visits was 33,142, of whom 7,527 patients were admitted; 197 patients deceased (128 died in the hospital and 69 died upon arrival), and 25,401 patients were discharged, of whom 360 had sought discharge against medical advice.

## Discussion

Ramadan is the holiest month for Muslims, during which the believers follow certain special practices that can have an impact on the individual's health and life. In this study, we aimed to analyze the data of patients who visited the ED during the month of Ramadan and the two lunar months that precede and succeed Ramadan (Shaban and Shawal) for the period spanning 2019-211. Our study revealed a gender variation regarding ED visits with a higher frequency of female visits across all three months. Youth and adults aged 19-39 years constituted nearly half of the visits. The frequency of visits by prepubertal children was lower, which could be attributed to the fact that fasting during Ramadan is only required for postpubertal individuals. Similar findings have been reported from other countries regarding the flow of patients during Ramadan [10,11]. When comparing our findings with those in the literature, minimal variation was observed in terms of patient volume; however, significant differences were observed with regard to the type of complaints; this is in line with the results of a study conducted in Riyadh, Saudi Arabia [12]. In contrast with our findings, a study performed at a tertiary hospital in Abu Dhabi reported a significant reduction in ED visits during Ramadan [10]. We observed a difference between the two countries (UAE and Saudi Arabia) in terms of the extent to which Ramadan changes people's lifestyles and their flexibility and comfort in breaking their fast in hospitals, where oral tablets or IV cannulas are easily available.

Our study analyzed the most common complaints among patients visiting the ED. Fever was the leading cause of ED admission, followed by abdominal pain, shortness of breath, cough, and chest pain. However, minimal variation was observed among other complaints, which included sore throat, headache, vomiting, fatigue, and dizziness, respectively. Also, Ramadan witnessed a similar pattern of complaints across the three years. As for analyzing each complaint individually, fever, which was generally the most common complaint, was not the most common complaint in 2019 during the three lunar months. A rebound of fever cases was observed in Shaban in 2020. The month of Shaban 2020 corresponded to March in the Gregorian calendar. On March 2, 2020, Saudi health authorities announced the first case of COVID-19 in the nation [13]. Subsequently, a decrease in ED visits for the specific disease was reported [14]. Saudi health authorities imposed restrictions in light of the pandemic, and religious pilgrimages to Makkah and Madinah were suspended, as were school and work attendance, and a quarantine system was imposed [15]. We believe that the fear of being infected in the hospital discouraged patients from visiting the ED unless they had symptoms of infection.

Pregnancy-related complications were the most common cause of ED visits in Shaban and Ramadan in 2019, but their numbers dropped during the rest of the study period. According to the Saudi Open Data portal, the rate of newborns was almost 400,000 during that period [16]. On the other hand, throughout 2020, which corresponded with the COVID-19 pandemic and quarantines being imposed [15], we found that the newborn rate increased by almost two times compared to 2019. However, pregnancy-related complaints were the first or second most common cause in Ramadan and Shawal in 2020. Even though the association between pregnancy and COVID-19 was not apparent at that time, we assume that restrictions imposed by the government left couples with fewer outdoor recreational options, which led them to stay indoors together for longer periods of time and provided more opportunities for occasions of intimacy.

This study documents that out of 33,142 patients who visited the ED, only 22.7% were admitted, while some other studies have recorded an admission rate of 45.8% between 2005 and 2008 [17]. Generally, it is normal for government-funded hospitals to experience a lower admission rate; this can be attributed to the lack of bed vacancies since patients receive treatment and care free of charge. This puts universal healthcare systems in a dilemma in ensuring justice and equity in terms of care provided to the patients, especially given that the rate of mortality and morbidity tends to increase with prolonged ED boarding [17]. Patients leaving against medical advice is one of the most prevalent healthcare-related issues worldwide, contributing to high rates of morbidity and mortality as well as a heavy financial burden on the healthcare system [18]. In our study, 1.09% of patients left against medical advice. Their reasons varied and were mainly due to their refusal to undergo procedures or operations, long ED waiting times, subjective improvement with treatment, and the need to return to children at home.

## Conclusions

The result of our study illustrates the change in ED visit patterns during the unique period of the study: the holy month of Ramadan and the months that precede and succeed it. Also, the type of complaints was strongly affected by the COVID-19 pandemic, which corresponded partly with the study period. In terms of patient outcomes, significant progress was reported following ED visits, which raised more questions about the reason behind these outcomes. We recommend more research in this field given the current lack of studies in the literature. The healthcare administration should consider using this time of year to redistribute resources and assign healthcare workers to serve the system more efficiently. Moreover, our findings highlight that studying the ED data can help provide accurate information that can be used to help modify/adjust the quality of services provided in the ED.

## References

[1] Fernando HA, Zibellini J, Harris RA, Seimon RV, Sainsbury A (2019). Effect of Ramadan fasting on weight and body composition in healthy non-athlete adults: a systematic review and meta-analysis. Nutrients.

[2] Lessan N, Ali T (2019). Energy metabolism and intermittent fasting: the Ramadan perspective. Nutrients.

[3] Roky R, Chapotot F, Hakkou F, Benchekroun MT, Buguet A (2001). Sleep during Ramadan intermittent fasting. J Sleep Res.

[4] Parnell JA, Reimer RA (2009). Weight loss during oligofructose supplementation is associated with decreased ghrelin and increased peptide YY in overweight and obese adults. Am J Clin Nutr.

[5] Patel SR, Hu FB (2008). Short sleep duration and weight gain: a systematic review. Obesity (Silver Spring).

[6] Elbarsha A, Elhemri M, Lawgaly SA, Rajab A, Almoghrabi B, Elmehdawia RR (2018). Outcomes and hospital admission patterns in patients with diabetes during Ramadan versus a non-fasting period. Ann Saudi Med.

[7] Shanks NJ, Ansari M, al-Kalai D (1994). Road traffic accidents in Saudi Arabia. Public Health.

[8] Riazul Haq and Shahbaz Rana (27 May 2018 (2023). Headcount finalised sans third-party audit. https://tribune.com.pk/story/1719994/headcount-finalised-sans-third-party-audit.

[9] AlKhaldi YM, AlKhaldi AY, AlQahtani AS, Al-Shahrani BS, Meshawi EA, Albishri BM (2019). Incidence of hypoglycemia and its risk factors among diabetics during Ramadan in Abha city, Aseer Region, KSA. J Family Med Prim Care.

[10] Faruqi I, Mazrouei LA, Buhumaid R (2020). Impact of Ramadan on emergency department patients flow; a cross-sectional study in UAE. Adv J Emerg Med.

[11] Al Assaad RG, Bachir R, El Sayed MJ (2018). Impact of Ramadan on emergency department visits and on medical emergencies. Eur J Emerg Med.

[12] Butt T, Khan HU, Ahmed I, Eldali A (2016). Emergency department attendance patterns during Ramadan. Ann Saudi Med.

[13] Nurunnabi M (2020). The preventive strategies of COVID-19 pandemic in Saudi Arabia. J Microbiol Immunol Infect.

[14] Baum A, Schwartz MD (2020). Admissions to Veterans Affairs hospitals for emergency conditions during the COVID-19 pandemic. JAMA.

[15] Rennert-May E, Leal J, Thanh NX (2021). The impact of COVID-19 on hospital admissions and emergency department visits: a population-based study. PLoS One.

[16] (2023). Birth statistics data. https://data.gov.sa/Data/ar/dataset/birth-statistics-data-for-the-year-1441-ah.

[17] Singer AJ, Thode HC Jr, Viccellio P, Pines JM (2011). The association between length of emergency department boarding and mortality. Acad Emerg Med.

[18] Abuzeyad FH, Farooq M, Alam SF (2021). Discharge against medical advice from the emergency department in a university hospital. BMC Emerg Med.

